# Mixotrophic Growth Under Micro-Oxic Conditions in the Purple Sulfur Bacterium “*Thiodictyon syntrophicum*”

**DOI:** 10.3389/fmicb.2019.00384

**Published:** 2019-03-05

**Authors:** Samuel M. Luedin, Nicola Storelli, Francesco Danza, Samuele Roman, Matthias Wittwer, Joël F. Pothier, Mauro Tonolla

**Affiliations:** ^1^Microbiology Unit, Department of Botany and Plant Biology, University of Geneva, Geneva, Switzerland; ^2^Laboratory of Applied Microbiology, Department of Environment, Constructions and Design, University of Applied Sciences of Southern Switzerland (SUPSI), Bellinzona, Switzerland; ^3^Spiez Laboratory, Biology Division, Federal Office for Civil Protection, Spiez, Switzerland; ^4^Alpine Biology Center Foundation, Bellinzona, Switzerland; ^5^Environmental Genomics and System Biology Research Group, Institute of Natural Resource Sciences, Zurich University of Applied Sciences (ZHAW), Wädenswil, Switzerland

**Keywords:** sulfur cycling, carbon fixation, meromixis, Lake Cadagno, mixotrophic growth, purple sulfur batceria

## Abstract

The microbial ecosystem of the meromictic Lake Cadagno (Ticino, Swiss Alps) has been studied intensively in order to understand structure and functioning of the anoxygenic phototrophic sulfur bacteria community living in the chemocline. It has been found that the purple sulfur bacterium “*Thiodictyon syntrophicum*” strain Cad16^T^, belonging to the Chromatiaceae, fixes around 26% of all bulk inorganic carbon in the chemocline, both during day and night. With this study, we elucidated for the first time the mode of carbon fixation of str. Cad16^T^ under micro-oxic conditions with a combination of long-term monitoring of key physicochemical parameters with CTD, ^14^C-incorporation experiments and quantitative proteomics using *in-situ* dialysis bag incubations of str. Cad16^T^ cultures. Regular vertical CTD profiling during the study period in summer 2017 revealed that the chemocline sank from 12 to 14 m which was accompanied by a bloom of cyanobacteria and the subsequent oxygenation of the deeper water column. Sampling was performed both day and night. CO_2_ assimilation rates were higher during the light period compared to those in the dark, both in the chemocline population and in the incubated cultures. The relative change in the proteome between day and night (663 quantified proteins) comprised only 1% of all proteins encoded in str. Cad16^T^. Oxidative respiration pathways were upregulated at light, whereas stress-related mechanisms prevailed during the night. These results indicate that low light availability and the co-occurring oxygenation of the chemocline induced mixotrophic growth in str. Cad16^T^. Our study thereby helps to further understand the consequences micro-oxic conditions for phototrophic sulfur oxidizing bacteria. The complete proteome data have been deposited to the ProteomeXchange database with identifier PXD010641.

## Introduction

Inorganic carbon, nitrogen and sulfur are cycled in diverse microbial metabolic networks in paired redox reactions as a result of which organic compounds are produced in the scale of 10^9^ t year^−1^ worldwide (Falkowski et al., 2008). In order to overcome the endergonic energy barrier in assimilatory processes, light is used as a source of energy in phototrophic anoxygenic bacteria since at least 3.85 Ga (Hohmann-Marriott and Blankenship, 2007). During inorganic carbon fixation, the transfer of electrons along a gradient of carrier molecules with sequentially lower potential allows the generation of energy bound to ATP and reductants [e.g., NAD(P)H and reduced ferredoxin]. The electrons required to replenish the oxidized electron acceptor pool can be derived from the oxidation of reduced sulfur species such as sulfide, sulfite and thiosulfate, or H_2_ and Fe (II) (Blankenship et al., 1995).

The anoxygenic photosynthetic purple sulfur bacteria (PSB) of the family Chromatiaceae are widespread in aquatic, sulfidic oxygen minimum-zones where light is still available (Imhoff, 2014). As an adaption to low light availability around 0.1–20 μmol quanta m^2^ s^−1^ and only limited wavelength (450–600 nm and infrared above 750 nm), PSB contain pigments of the carotenoid and bacteriochlorophyll *a* and *b* (BChl) classes (Pfennig et al., 1968), as well as multiple copies of antenna peptides, to subtly modulate charge separation within the membrane bound type II reaction center (Wagner-Huber et al., 1992; Weissgerber et al., 2011). Carbon is typically fixed through the Calvin-Benson-Bassham (CBB) cycle (Tabita, 1988). In order to store both, reduction-equivalents and oxidized carbon, PSB intracellularly concentrate elemental sulfur-chains (S-${\text{S}}_{\text{n}}^{0}$) in protein covered globules (Prange et al., 2002), as well as glycogen and polyhydroxybutyrate (PHB) (Schlegel, 1962), respectively. These assimilates may subsequently allow for chemotrophic growth in the dark (Gemerden, 1968; Del Don et al., 1994; Storelli et al., 2014).

The possibility of chemolithoautotrophic growth of PSB under microaerophilic conditions in the dark has been proposed by van Niel (1941) and has been described first for PSB *Thiocapsa roseopersicina* istrain BBS (Bogorov, 1974). *Thiocapsa roseopersicina* inhabiting shallow tidal flats is especially adapted to the daily changes of oxygen concentration and competes with chemolithotrophic colorless sulfur bacteria (*Thiobacillus* spp.) and *Beggiatoaceae* spp. (de Wit and van Gemerden, 1987). Chemoheterotrophic, chemoautotrophic and mixotrophic growth has since been shown for different PSB spp. (Hurlbert, 1967; Kondratieva et al., 1976; Kämpf and Pfennig, 1986; Overmann and Pfennig, 1992; Schaub and van Gemerden, 1994; Rákhely et al., 2007). Several strains of *Allochromatium vinosum* and *T. roseopersicina* have shown mixotrophic growth under a 5% oxygen atmosphere with acetate and reduced sulfur compounds (Kämpf and Pfennig, 1986).

The ecological significance and the impact on biogeochemistry of PSB have been extensively studied in permanently stratified lakes (Genovese and Trüper, 1968; Sorokin, 1970; Schanz et al., 1998; Tonolla et al., 1999; Hamilton et al., 2014; Pjevac et al., 2015). In Lake Cadagno (Piora valley, Swiss Alps), underwater springs in gypsum rich dolomite provide a steady inflow of solute-rich water. In combination with solute-poor surface water, a stable and steep gradient in redox potential, salinity, sulfide and oxygen concentrations at around 12 m depth is formed (Del Don et al., 2001). Within this chemocline, a dense population of phototrophic sulfur oxidizing bacteria of the family Chromatiaceae and Chlorobiaceae (GSB; green sulfur bacteria) annually thrive to dense populations with up to 10^7^ cells mL^−1^ between June and October (Tonolla et al., 2017). *In situ* chemocline incubation experiments with ^14^C-uptake in Lake Cadagno (Camacho et al., 2001; Storelli et al., 2013), and other lakes (Casamayor et al., 2008), as well as with single-cell HISH-SIMS [halogen *in situ* hybridization secondary-ion mass spectroscopy] (Musat et al., 2008) revealed both light-driven and dark carbon fixation of PSB. It was found that the population of PSB isolate “*Thiodictyon syntrophicum*” sp. nov. str. Cad16^T^ (str. Cad16^T^, thereafter) (Peduzzi et al., 2012) assimilated 26% of the total carbon in the chemocline during dark and light incubations (Storelli et al., 2013). Alternatively, the relative contribution of different PSB and GSB spp. to total carbon assimilation normalized to biomass during daytime was estimated with a C-isotope fractionation study for Lake Cadagno. There it was found that str. Cad16^T^ fixed photosynthetically only 1.3–2% of the total carbon, as estimated from the daily bulk δ^13^C-mass balance (Posth et al., 2017).

Additional insight came from an *in vitro* quantitative proteomics study with str. Cad16^T^ growing anaerobically under light and dark conditions with 1 mM H_2_S (Storelli et al., 2014). Photosynthesis-driven growth of str. Cad16^T^ resulted in the relative >1.5× expression of 22 proteins. Most notably, the poly(R)-hydroxyalkanoicacid synthase subunit PhaE and the phasin PhaP involved in the synthesis of PHB were found. In contrast, among the 17 proteins overexpressed under dark conditions, three enzymes of the dicarboxylate/4-hydroxybutyrate (DC/HB) cycle were detected. However, the absence of the complete set of DC/HB cycle genes in the genome of str. Cad16^T^ indicates that these enzymes are rather involved in the reverse tricarboxylic acid cycle and the last step of the beta-oxidation of fatty acid and PHB granules (Luedin et al., 2018a). The complete genome of str. Cad16^T^ gave evidence of the biological functions encoded (Luedin et al., 2018a). Similar to *Allochromatium* spp. or *Lamprocystis* spp., str. Cad16^T^ expresses a type II (quinone type) reaction center, the membrane-bound protein cascade of cyclic electron transport to generate ATP and reverse electron transport to produce NAD(P)H, and also contains a *cbb*3 type cytochrome *c*. The latter may enable microaerobic growth and Fe(III) oxidation of str. Cad16^T^ (Berg et al., 2016). However, no genetic evidence for the possible syntrophic relationship within aggregates of *Desulfocapsa* sp., and also only incomplete Sox and no thiosulfate dehydrogenase Tsd proteins, responsible for ${\text{SO}}_{3}^{2-}$ oxidation, as previously described for str. Cad16^T^ by Peduzzi et al. (2012) were found.

With this study, we aimed at elucidating the microaerobic metabolic mechanisms of PSB in the presence and absence of light, using str. Cad16^T^ as model organism. The main objectives of this study were: (i) to study differences between day and night in the metabolism of PSB str. Cad16^T^ incubated *in situ* at 12 m in the chemocline of Lake Cadagno and (ii) to monitor the environmental factors longitudinally that could influence the metabolic activity of the chemocline community. In order to quantify differences in metabolic activity, we used a combination of CO_2_ assimilation analysis using radioactive isotope ^14^C and label-free quantitation tandem mass spectrometry (LFQ-MS^2^) based proteomics. Relative light intensity and temperature at the depth of the chemocline were measured constantly and several physicochemical profiles were taken during the period of incubation. The unique micro-oxic *in situ* conditions measured in summer 2017 were reflected in the carbon assimilation rates and protein profiles obtained and revealed mixotrophy in str. Cad16^T^.

## Materials and Methods

### Study Site and Field Measurements

The *in situ* incubations were performed in Lake Cadagno between 13 July 2017 to 12 September 2017 with dialysis bags attached to a mooring (46°,33′,05,1′′ N/8°,42′,43,0′′ E, max. depth 18 m) (Figures S1A,B). From 13 July to 13 September 2017 different physical and chemical parameters were measured alongside the incubations in order to monitor the *in situ* chemocline conditions and adjust the incubation depth, if necessary. A CTD 115 probe (Sea & Sun Technology GmbH, Germany) equipped with temperature, salinity, oxygen and turbidity sensors was used to measure physicochemical profiles from a working platform at the anchor site of the mooring. Profiles from 13 July 2017 were taken as an estimate of chemocline depth (Figure S2). HOBO UA-002-64 Pendant passive data loggers (Onset Computer Corporation, MA, USA) measured relative light (Lux; 180–1,200 nm) and temperature at 60 min intervals. Two sensors were placed near the surface (0.05 m depth) and other pairs were positioned 0.4 m apart at the upper and lower part of the rig, respectively (Figure S1B). Empirical conversion factors of 1 Lux = 0.018 PAR and 0.016 PAR (μmol quanta m^−2^ s^−1^) were used for the surface and below the water, respectively as in Thimijan and Heins (1983). The HOBO logger values were analyzed after their retrieval at the end of the experiment. The Alpine Biology Center Foundation has research permission for Lake Cadagno from the cantonal government of Ticino (Switzerland).

We additionally had access to CTD data from a parallel project from Oscar Sepúlveda Steiner and colleagues from EAWAG (Dübendorf, Switzerland) where two CTD profiles were taken daily.

### Flow Cytometry for Cell Counting

Flow-cytometry (FCM) based cell counting was performed as in Danza et al. (2018). In short, phototrophic bacteria were identified using 50 μl sub-samples in triplicates with a BD Accuri C6 cytometer (Becton Dickinson, San José, CA, USA). The flow rate was set to 66 μL min^−1^.The analysis was performed with the BD Accuri C6 Plus software (Becton Dickinson, San José, CA, USA, RRID:SCR_014422). Two lasers (488 and 680 nm), two scatter detectors, and two fluorescence detectors (FL3 = 670 nm and FL4 = 670) were used. To exclude abiotic particles a forward scatter threshold of FSC-H 10,000 was applied. A red fluorescent (FL3) threshold above 1,100 was applied to select for cells emitting autofluorescence due to Chl and BChl.

### Estimates of Carbon Uptake Rates

Estimations on microbial carbon uptake from previous studies were considered in our analysis. Daily carbon uptake estimations for anoxygenic and oxygenic photosynthesis for Lake Cadagno were conducted by Camacho et al. (2001). It was found that half of the carbon is fixed through oxygenic and anoxygenic photosynthesis, respectively. Musat and colleagues estimated anoxygenic carbon assimilation for PSB *C. okenii* and GSB *C. clathratiforme* to be 70 and 15% of the total daily CO_2_ photo-assimilation, respectively (Musat et al., 2008). We therefore calculated the uptake rates for the three populations as follows (Equation 1) where *A*_day_ is the total uptake rate per cell of the phototrophic community and *F*_*popN*_ the fraction as estimated during the day in Musat et al. (2008) and for the night in Storelli et al. (2013) for population N, respectively:

(1) $$\begin{array}{r} A_{popN}=\;A_{day}\times 0.5\times F_{popN} \end{array}$$

### Bacterial Pure Cultures and Dialysis Experiment

Str. Cad16^T^ was isolated in 2003 (Peduzzi et al., 2003) and was subsequently grown in pure culture on autotrophic Pfennig's medium II (Eichler and Pfennig, 1988) at the Laboratory of Applied Microbiology (LMA) in Bellinzona, Switzerland (Figure S1C). The medium contained 0.25 g of KH_2_PO_4_ L^−1^, 0.34 g of NH_4_Cl L^−1^, 0.5 g of MgSO_4_·7H_2_O L^−1^, 0.25 g of CaCl_2_·H_2_O L^−1^, 0.34 g of KCl L^−1^, 1.5 g of NaHCO_3_ L^−1^, 0.02 mg of vitamin B12 L^−1^ and 0.5 mL of trace element solution SL10 L^−1^. The medium was autoclaved under a 80% N_2_ /20% CO_2_ atmosphere (Widdel and Bak, 1992) and 1.1 mM Na_2_S·9H_2_O was added aseptically. The pH was adjusted to 7.0. Cultures were grown in 500 mL glass bottles at ambient temperature and under a 12/12 h light/dark-regime with a 60 W incandescent lamp (6 μmol quanta m^−2^ s^−1^). Cells were grown up to a concentration of around 3 × 10^6^ cells mL^−1^. Cell concentrations repeatedly were measured by flow cytometry.

Cellulose dialysis bags with a 14 kDa cutoff (D9777-100FT, Sigma-Aldrich, Buchs, CH) were rinsed for 1.5 h in Na_2_CO_3_ (40 g L^−1^) and 0.01 M EDTA at 60°C while stirring. The bags were cleaned with ddH_2_O, cut into 0.6 m long pieces, closed by a knot on one end and autoclaved for 20 min at 121°C. On site, about 80 mL of bacteria culture (3 × 10^6^ cells mL^−1^) were filled randomly in each bag, which was closed, attached to a rig and installed in the chemocline within 30 min (Figure S1B). In total 18 dialysis bags were placed at 12 m depth from 13 July to 23 August 2017 and then lowered to 14 m for the remaining campaign.

### ^14^C-Incubations

The ^14^C-radioisotope uptake experiment was performed as in Storelli et al. (2013). In short, volumes of subsamples from three dialysis bags were pooled together randomly, 7 mL NaH^14^CO_3_ (NaH^14^CO_3_; 1.0 mCi; 8.40 mCi mM L^−1^, 20 μCim L^−1^; Cat. No. NEC-086S Perkin-Elmer, Zurich, Switzerland) were added and thereof, six technical replicates were transferred to 50 mL translucent Duran glass bottles (SCHOTT AG, Mainz, Germany). The bottles were then incubated at a depth of 14 m in a mesh basket on the rig for 4 h during day (1:00–4:00 p.m.) and night (9:00 p.m.−12:00 a.m.), respectively. Chemocline background fixation rates were determined in 50 mL chemocline samples. Filtered chemocline lake water (0.45 μm) was used as negative control. Upon retrieval, the amount of β-activity (^14^C) assimilated by microbes during the incubation time was measured in the laboratory following standard method that included acidification and bubbling of the samples (Gächter et al., 1984).

Scintillation was done on a *Guardian 1414* liquid scintillation counter (Perkin Elmer Wallac, MA, USA) running with the WinSpectral v.1.40 software. Raw data was statistically analyzed using *t*-tests in Excel (Microsoft Office 2010, v-0.14.0.7168.5000).

The inorganic dissolved carbon concentration was determined with the CaCO_3_ *Merck Spectroquant* kit No. 1.01758.0001 and the *Merck spectroquant Pharo 100* photospectrometer (Merck & Cie, Schaffhausen, Switzerland). Samples were taken at 14 m depth, filtered with 0.45 μm filters, pH was tested with indicator paper (MColorpHast, Merck KGaA, Germany) to lie within 6.8–7.0 and triplicate samples were measured.

### Protein Extraction and Digest

Equal volumes of subsamples were transferred to 50 mL tubes upon retrieval and stored at 4°C in the dark. They were then brought to the lab within 30 min and centrifuged 10,000 g at 4°C for 10 min. The supernatant was discarded and the pellets were re-suspended in 1× PBS pH 7.0 and 1% EDTA-free Protease Inhibitor Cocktail (*v/v*; Thermo Fisher Scientific, Reinach, Switzerland) and frozen at −20°C until further processing.

The cells were thawed, lysed in 5% sodium-deoxycholate (w/w) in 100 mM ammonium-bicarbonate buffer containing 1% EDTA-free Protease Inhibitor Cocktail (*v/v*; Thermo Fisher Scientific, Reinach, Switzerland) and sonicated for 15 min at 200 W at 10°C with a Bioruptor ultrasonicator (Diagenode SA, Belgium). Samples were then shipped to the Functional Genomic Center Zurich (FGCZ) on dry ice for further processing. Protein concentration was estimated using the Qubit Protein Assay Kit (Thermo Fisher Scientific, Reinach, Switzerland). The samples were then prepared using a commercial iST Kit [PreOmics, Germany (Preomics-sample-prep-kit-protocol-cartridge-96x-v_3_0_b.pdf, 2018)] with an updated version of the protocol. Briefly, 50 μg of protein were solubilized in ‘lyse’ buffer, boiled at 95°C for 10 min and processed with High Intensity Focused Ultrasound (HIFU) for 30 s setting the ultrasonic amplitude to 85%. Then the samples were transferred to a cartridge and digested by adding 50 μl of the ‘Digest’ solution. After incubation (60 min, 37°C) the digestion was stopped with 100 μl of Stop solution. The solutions in the cartridge were removed by centrifugation at 3,800 g, while the peptides were retained by the iST-filter. Finally the peptides were washed, eluted, dried and re-solubilized in “LC-Load” buffer for Tandem Mass spectrometry (MS^2^)-analysis.

### Liquid Chromatography and MS^2^-Analysis

MS^2^ analysis was performed on a *QExactive* mass spectrometer coupled to a nano *EasyLC 1000* HPLC (Thermo Fisher Scientific, Reinach, Switzerland). Initial solvent composition was 0.1% formic acid for channel A and 0.1% formic acid, 99.9% acetonitrile for channel B, respectively. For each sample 4 μL of peptides were loaded on a commercial Acclaim *PepMap* Trap Column (75 μm × 20 mm; Thermo Fisher Scientific, Rheinach, Switzerland) followed by a *PepMap RSLC* C18 Snail Column (75 μm × 500 mm; Thermo Fisher Scientific, Reinach, Switzerland). The peptides were eluted at a flow rate of 300 nL min^−1^ by a gradient from 5 to 22% B in 79 min, 32% B in 11 min, and 95% B in 10 min. Samples were acquired in a randomized order. The mass spectrometer was operated in data-dependent mode (DDA), acquiring a full-scan MS spectra (300–1,700 *m/z*) at a resolution of 70,000 at 200 *m/z* after accumulation to a target value of 3,000,000, followed by HCD (higher-energy collision dissociation) fragmentation on the twelve most intense signals per cycle. HCD spectra were acquired at a resolution of 35,000, using a normalized collision energy of 25 a. u. and a maximum injection time of 120 ms. The automatic gain control (AGC) was set to 50,000 ions. Charge state screening was enabled and singly and unassigned charge states were rejected. Only precursors with intensity above 8,300 were selected for MS^2^ (2% underfill ratio). Precursor masses previously selected for MS^2^ measurement were excluded from further selection for 30 s, and the exclusion window was set at 10 ppm. The samples were acquired using internal lock mass calibration on *m/z* 371.101 and 445.120.

### Protein Identification and Label Free Protein Quantification

The acquired raw MS^2^ data were processed by MaxQuant v.1.4.1.2 (RRID:SCR_014485), followed by protein identification using the integrated Andromeda search engine. Each file was kept separate in the experimental design to obtain individual quantitative values. Spectra were searched against a forward str. Cad16^T^ database (6,237 coding genes), concatenated to a reversed decoyed *fasta* database and common protein contaminants (NCBI Assembly No. ASM281377v1; release date: 2017/12/07). Carbamidomethylation of cysteine was set as fixed modification, while methionine oxidation and N-terminal protein acetylation were set as variable. Enzyme specificity was set to trypsin/P allowing a minimal peptide length of 7 amino acids and a maximum of two missed-cleavages. Precursor and fragment tolerance was set to 10 ppm and 0.05 Da for the initial search, respectively. The maximum false discovery rate (FDR) was set to 0.01 for peptides and 0.05 for proteins. Label free quantification was enabled and for the quantification across multiple run-files the “match between runs” option with a retention time window of 2 min was enabled. The re-quantify option was selected. For protein abundance, the intensity (Intensity) as expressed in the protein groups file was used, corresponding to the sum of the precursor intensities of all identified peptides for the respective protein group. Only quantifiable proteins (defined as protein groups showing two or more razor peptides) were considered for subsequent analyses. Protein expression data were transformed (hyperbolic arcsine transformation) and missing values (zeros) were imputed using the *missForest R*-package v.1.4 (Stekhoven and Bühlmann, 2012). The protein intensities were normalized by scaling the median protein intensity in each sample to the same values.

Scaffold v.4.8.4 (Proteome Software Inc., Portland, OR, RRID:SCR_014345) was used to validate MS^2^ based peptide and protein identifications. Peptide identifications were accepted if they could be established at >42.0% probability to achieve an FDR <0.1% by the Peptide Prophet algorithm with *Scaffold* (Keller et al., 2002) delta-mass correction. Protein identifications were accepted if they could be established at >54.0% probability to achieve an FDR <1.0% and contained at least two identified peptides. Protein probabilities were assigned by the *Prophet* algorithm (Nesvizhskii et al., 2003). Proteins that contained similar peptides and could not be differentiated based on MS^2^ analysis alone were grouped to satisfy the principles of parsimony. Proteins sharing significant peptide evidence were grouped into clusters. For the two-group analysis the statistical testing was performed using (paired) *t*-test on transformed protein intensities (hyperbolic arcsine transformation). Proteins were called significantly differentially expressed if linear fold-change exceeded Two-fold and the *q*-value from the *t*-test was below 0.01.

As an alternative method to find differentially expressed proteins, we used the correlation adjusted *t*-Score algorithm provided by the *R*-package sda v.1.3.7 (Ahdesmäki and Strimmer, 2010) to further analyze the dataset of 1,333 proteins identified with MaxQuant.

### Protein Functional Annotation

BlastKOALA v.2.1 (Kanehisa et al., 2016, RRID:SCR_012773) and eggNOG v.4.5.1 (Huerta-Cepas et al., 2016, RRID:SCR_002456) were used to classify the proteins into functional categories. The complete KEGG-dataset for str. Cad16^T^ can be found under KEGG PATHWAY^1^ (Metabolic pathways—Candidatus *Thiodictyon syntrophicum*).

### Genomic Data Availability

The complete genome of str. Cad16^T^ (Luedin et al., 2018a) is available under the GenBank assembly GCA_002813775.1.

### Proteomic Data Availability

The complete proteomic data of str. Cad16^T^ have been deposited to the ProteomeXchange Consortium (ProteomeXchange Datasets^2^) via the PRIDE partner repository (Vizcaíno et al., 2012) under the accession PXD010641 and project DOI 10.6019/PXD010641.

## Results

### Physicochemical Parameters From July to August 2017

**Figure 1:**
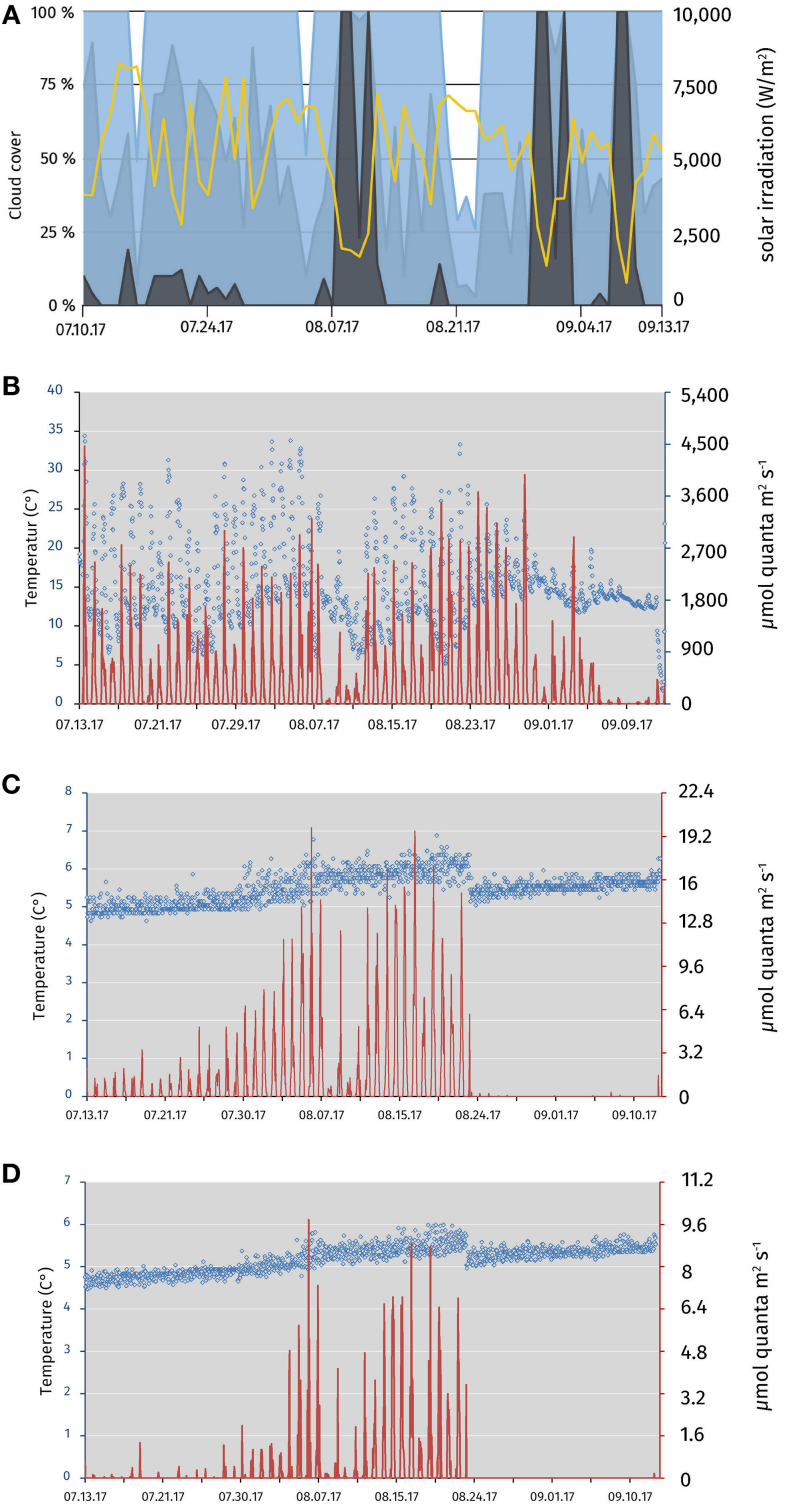
Meteorological data for the Piora valley, temperature, and relative light availability at different depths of the mooring in Lake Cadagno from July 13 to September 13 2017. **(A)** Sun light and cloud cover graph for the Piora valley from July 13 to September 13 2017. Data from meteoblue.com (yellow; sum of the daily shortwave radiation in W m^−2^, blue; maximal daily cloud cover in%, light blue; mean daily cloud cover in%, gray; minimal daily cloud cover in %). **(B)** Temperature (blue circles) and average light available at midnight/mid-day (red line) at the surface buoy of Lake Cadagno. The data logger was partly immersed in water, dampening the light and temperature readings (see values in August). **(C)** Relative light availability and temperature profile at midnight/mid-day of the two HOBO loggers at the top of the rig at 12 (July 13–August 23 2017) and 14 m depth (August 23–September 13 2017), respectively. A steady increase in average temperature and light availability from July to August is visible. The increase in the available light can be explained through the downward movement of photosynthetic bacteria over the season (see Figure S4) Re-positioning of the rig on the August 23 2017 results in both, a drop in temperature, and light availability. Available light was reduced to an average of 0.4 μmol m^−2^ s^−1^. **(D)** Relative light availability and temperature profile at midnight/mid-day of the two HOBO loggers at the bottom of the rig at 12.4 (July 13–August 23 2017) and 14.4 m depth (August 23–September 13 2017), respectively. Low light availability and temperature are characteristic for the depth of around 12.4 m. As in **(C)**, temperature and available light values are steadily rising from July to August. No light was detected during daytime at 14.4 m depth after repositioning in August. Data was logged in hourly intervals for **(B–D)**.

Fluctuations in the relative light intensity were positively correlated with the measured surface radiation and negatively associated with cloud cover (Figure 1A). Daily relative average surface light intensity at 12:00 p.m. was 1448.9 μmol quanta m^−2^ s^−1^ (4.3–3769.6; Figure 1B). The surface temperature was stable at an average of 15°C. High temperatures up to 40°C were measured due the solar heating of the logger casing (Figure 1B). The accumulated sunlight was above average for the weeks observed from (Figure S3). From 13 July to 23 August 2017 at 12 m depth, an average of 3.7 μmol quanta m^−2^ s^−1^ (0.2–26.2) was measured. At 12.4 m depth, a mean of 1.3 μmol quanta m^−2^ s^−1^ (0.04–7.8) was registered (Figures 1C,D). Within this period, changes of turbidity, oxygen and Chl *a* profiles in the daily CTD profiles indicated that the chemocline had been sinking from 12 to 13–14.5 m depth (Figure S4). To ensure chemocline conditions for the incubations, we adjusted the depth of the rig from 12 to 14 m depth at 23 August 2017. However, this resulted in an overall reduced relative light intensity at the position of the rig at 14 m for the days 23 August to 12 September 2017. Only an average of 0.4 μmol quanta m^−2^ s^−1^ (0.2–4.0) was measured at 14 m depth from 24 August to 13 September 2017, and no light was measured at 14.4 m depth after 23 August 2017. Temperature was stable around 5°C (4.6–6.1) at both incubation depths with a positive trend over the months (Figures 1C,D).

### Chemical and Physical Analysis of Lake Cadagno at Sampling Date

**Figure 2:**
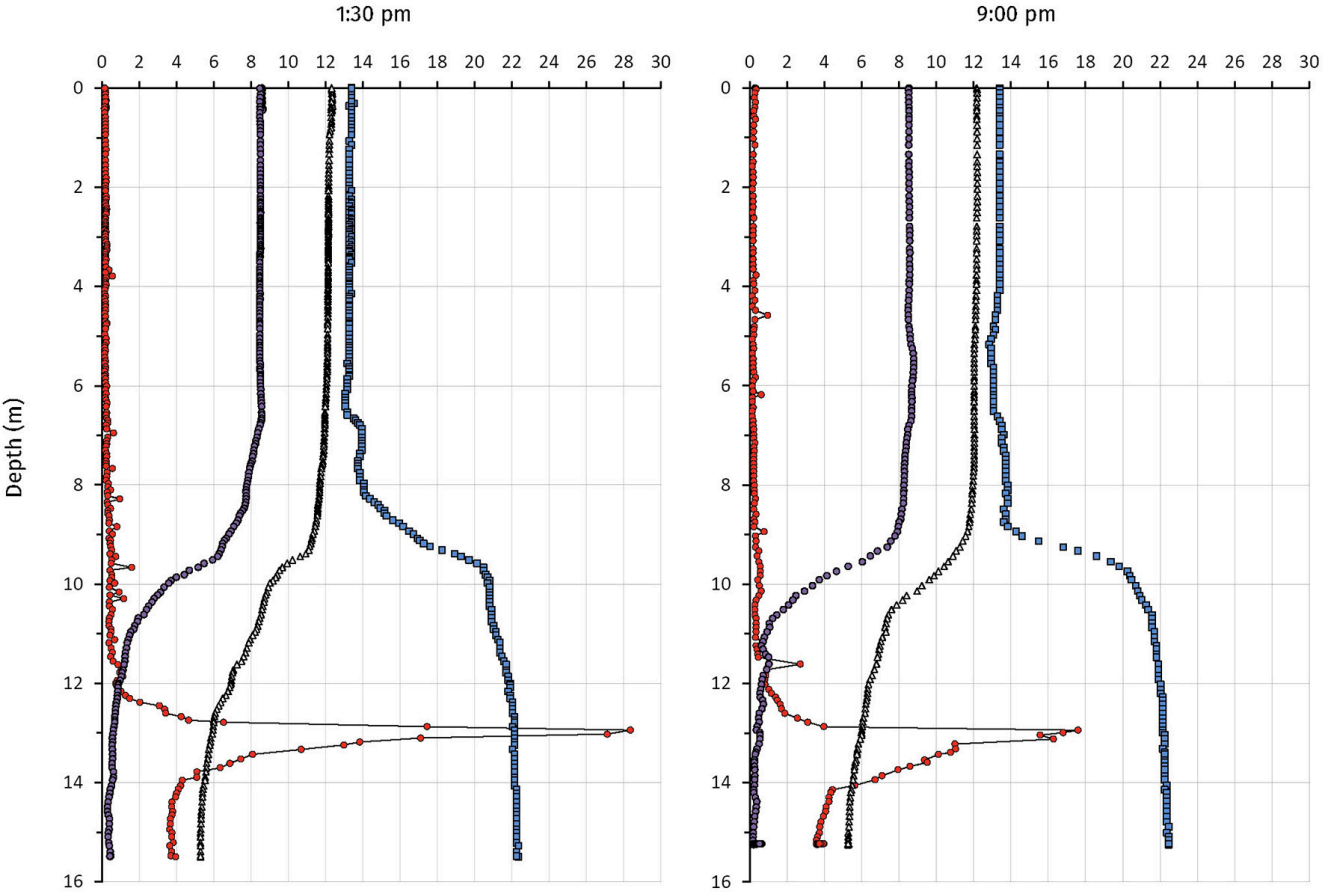
Profiles of physicochemical parameters from Lake Cadagno on September 12 (1:30 p.m.) and (9:00 p.m.) 2017. Measurements were taken from the platform. The CTD probe was equilibrated for 5 min at 0.5 m depth before measuring. Temperature (Δ), FTU, Formazin Turbidity Unit (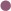); Cond, Conductivity (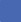); DO, Dissolved Oxygen (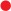).

Physicochemical measurements and endpoint-sampling was done on the 12 and 13 September 2017. The weather was cloudless with weak wind. Two CTD profiles at 1:30 p.m. and 9:00 p.m. showed a comparable situation for temperature, dissolved oxygen and conductivity (Figure 2). Water temperature was stable at 12°C from the surface down to the thermocline at 9.5 m, whereas it dropped to 5°C at 16 m depth at both time points. Dissolved oxygen (DO) was measured at 8.5 mg L^−1^ (265.6 μM) throughout the mixolimnion. From 7 to 9.5 m depth, DO-concentrations steadily declined to 6 mg L^−1^, and then more rapidly to 2 mg L^−1^ at 10.5 m depth, at both time points measured. At 14 m depth, 0.4 mg L^−1^ (12.5 μM) and 0.3 mg L^−1^ (9.4 μM) dissolved oxygen were measured during day and night, respectively. Conductivity increased along the profile from 0.13 in the mixolimnion to 0.22 mS cm^−1^ hypolimnion, both at day and night. In contrast, a pronounced turbidity peak (28 FTU) was observed at a depth of 13 m at 1:30 p.m. whereas a broader distribution of the FTU values (6–16 FTU) from 13 to 14 m depth, with a maximal peak of 18 FTU at 13 m, was observed at 9:00 p.m. Water samples taken at 1:30 p.m. and 9:00 p.m. from 14 m depth showed a milky and pink coloration, characteristic for a concentrated PSB at high FTU values. The total inorganic dissolved carbon concentrations measured at 14 m depth were 1.26 mM at 1:30 p.m. and 1.46 mM 9:00 p.m.

### Microbial Counts and Evaluation

Initial str. Cad16^T^ cell concentrations were on average 3.1 × 10^6^ cells mL^−1^ in July, as measured by flow cytometry. The rigged cultures were checked on 23 August 2017 and all dialysis bags were found intact and cells were judged healthy due to the turbid-pinkish appearance. When retrieved for sampling on 12 September 2017, all dialysis bags were still intact, the population was uniformly distributed within the dialysis bags and the cells grew to a mean concentration of 9.3 × 10^6^ cells mL^−1^. In total, the number of str. Cad16^T^ cells increased three-fold from July to September.

The average concentration of phototrophic cells in the lake sample taken at 14 m (12 September 2017) was 4.23 × 10^5^ cells mL^−1^ at 1:30 p.m., whereas it was 2.73 × 10^5^ cells mL^−1^ at 9:00 p.m., corresponding to 33.4 and 23.5% of the total events count by FCM, respectively. Moreover, FCM revealed that the phototrophic microbial community at 1:30 p.m. consists mainly of *C. okenii, Chlorobium* sp., and cyanobacteria spp. with 1.31 × 10^5^, 1.49 × 10^5^, and 1.57 × 10^5^ cells mL^−1^, representing 10.3, 11.8, and 12.4% of the total cell counts, respectively. At 9:00 p.m. the population at 14 m was found to consist of 5.37 × 10^4^ cells mL^−1^ *C. okenii*, 9.82 × 10^4^ cells mL^−1^ *Chlorobium* sp. and 1.03 × 10^5^ cells mL^−1^ with 4.6, 8.5, and 8.9% of all phototrophic cells, respectively. Together *C. okenii* and *Chlorobium* sp. corresponded to the 66.1 and 55.7% of the total anoxygenic phototrophic sulfur bacteria cells, during the day and at night, respectively. No significant difference in total cell concentration and internal complexity was measured between the two sampling groups (*P* = 0.74).

### *In situ* Carbon Fixation Rates

**Figure 3:**
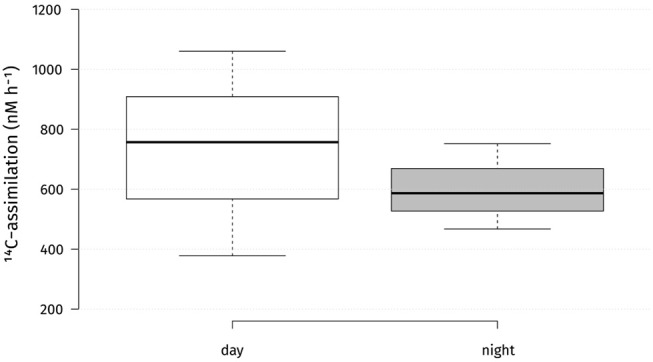
Absolute microbial ^14^C-uptake rates in the chemocline of Lake Cadagno during day and night. Center lines show the medians; box limits indicate the 25th and 75th percentiles as determined by *R* software; whiskers extent 1.5 times the interquartile range from the 25th and 75th percentiles. *n* = 3 sample points.

**Figure 4:**
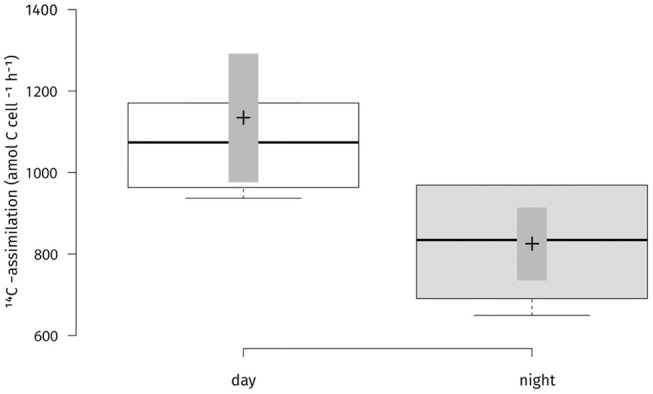
Carbon uptake rates per cell for “*Thiodictyon syntrophicum*” strain Cad16^T^ cultures for two conditions (day/night) during 4 h incubation *in situ*. ^14^C-radioisotope uptake experiments were performed on six biological replicates. Two side *t*-test statistics was applied. The difference in uptake rates between the two conditions was statistically significant at *p* < 0.05 (*p* = 0.02). Center lines show the medians; box limits indicate the 25th and 75th percentiles as determined by *R* software; whiskers extent 1.5 times the interquartile range from the 25th and 75th percentiles; crosses represent sample means; bars indicate 83% confidence intervals of the means; data points are plotted as open circles. *n* = 6 sample points.

Absolute carbon fixation rates in the chemocline at 14 m were comparable between day and night when tested, with medians of 757 and 587 nM h^−1^, respectively (Figure 3). During the light period, PSB *C. okenii*, GSB *Chlorobium* spp. and cyanobacteria assimilated in average 2,023, 381, and 2,417 amol cell^−1^ h^−1^, respectively (Figure S5). For the night fixation rates we used the estimates from Storelli et al. (2013). During the night, *C. okenii* possibly fixed up to 7,652 and GSB *Chlorobium* spp. around 84 amol C cell^−1^ h^−1^ , respectively (Figure S5). Str. Cad16^T^ fixed carbon in both conditions of light or dark (Figure 4). For str. Cad16^T^ ^14^C-bicarbonate median uptake rates normalized per cell were significantly different between the conditions with 1,074 amol C cell^−1^ h^−1^ (range: 937–1,585) during the day, and 834 amol C cell^−1^ h^−1^ (range; 650–969) during the night (Figure 4).

### Total Proteins Identified With LC-MS^2^-LFQ

A total of 11 samples, five samples for light period and six samples for the dark period, were processed for quantitative bottom-up proteomics and protein quantification was performed using the MaxQuant package. We used corrected *t*-test based statistics in order to identify and quantify proteins. The samples Cad16T_dia_7 (“light”) and Cad16T_dia_12 (“dark”) were identified as outliers in cluster analysis and were excluded from further data analysis. Therefore, the data analysis was made with four samples of the category “light” and five samples of the category “dark.” Overall a total of 1,333 proteins—21% of the total protein coding sequences—with at least two peptides were identified.

### Proteins Quantified With LC-MS^2^-LFQ

Peptide identifications were accepted if they could be established at >42.0% probability to achieve a false discovery rate (FDR) <0.1% with Scaffold delta-mass correction, resulting in 12,576 spectra included. Protein identifications were accepted if they could be established at >54.0% probability to achieve a FDR <1.0% and contained at least two identified peptides. The number of quantified proteins per condition was similar, with an average of 374 for the day and 354 for night period, respectively. Between 102 and 663 proteins were quantified in each biological replicate (7.2–37% of all IDs). Consequently, 684 proteins were quantified over all samples. Thereof, 21 contaminants were excluded. The remaining 663 proteins were classified with blastKOALA and EggNOG, with 627 annotated proteins for (99%) COG and 460 annotated proteins (69.4%) for blastKOALA, respectively.

As expected, many of the proteins with unchanged abundance belonged to the functional categories energy conversion, genetic information processing, carbohydrate and amino metabolism and protein modification (Table 1). Among the most abundant proteins detected were F_0_F_1_ ATPase subunits (AUB84561.1, AUB81565.1, AUB81565.1, and AUB84563.1) and chaperons (GroEL; AUB81575.1, AUB80010.1, AUB84066.1, DnaK; AUB84026.1). Since cell growth depends on protein synthesis, we found 36 ribosomal subunits as well as elongation factor Tu (AUB80476.1) to be equally abundant. The cells always contained the established components of the dissimilatory sulfide oxidation pathway such as ATP-sulfurylase Sat (AUB82369.1), the adenylylsulfate reductase AprAB (AUB82371.1, AUB82370.1), and the dissimilatory sulfite reductase (Dsr) complex (AUB83448.1–AUB83455.1). A Sqr sulfide:quinone reductase and a glutathione amide reductase GarA homolog to *A. vinosum* putatively involved in intracellular sulfur shuttling (Frigaard and Dahl, 2008) were also present. In PSB, sulfur oxidation provides the electrons for cyclic electron transport driven by light. Consequently, in str. Cad16^T^ PufMCL (AUB85378.1–AUB85380.1) and PuhA (AUB85431.1) subunits forming the reaction center II and six different PufAB antenna proteins (AUB85355.1–AUB85357.1, AUB85363.1, AUB85361.1, AUB85710.1) were expressed.

**Table 1:** Functional categories of “*Thiodictyon syntrophicum*” strain Cad16^T^ proteins that were identified with LC-MS^2^.

**COG code**	**Description**	**No. entries**
J	Translation, ribosomal structure, and biogenesis	64
K	Transcription	15
L	Replication, recombination and repair	7
D	Cell cycle control, Cell division, chromosome partitioning	5
V	Defense mechanisms	3
T	Signal transduction mechanisms	19
M	Cell wall/membrane biogenesis	28
N	Cell motility	1
U	Intracellular trafficking and secretion	10
O	Posttranslational modification, protein turnover, chaperones	50
C	Energy production and conversion	93
G	Carbohydrate transport and metabolism	35
E	Amino acid transport and metabolism	52
F	Nucleotide transport and metabolism	12
H	Coenzyme transport and metabolism	27
I	Lipid transport and metabolism	18
P	Inorganic ion transport and metabolism	37
Q	Secondary metabolites biosynthesis, transport and catabolism	6
**KEGG FUNCTIONAL CATEGORY**		
Genetic information processing		78
Carbohydrate metabolism		78
Energy metabolism		41
Metabolism of cofactors and vitamins		29
Amino acid metabolism		28
Environmental information processing		27
Nucleotide metabolism		17
Metabolism of terpenoids and polyketides		11
Lipid metabolism		9
Metabolism of other amino acids		5

EggNOG v.4.5.1 (Huerta-Cepas et al., 2016) and blastKOALA v.2.1 (Kanehisa et al., 2016) were used to classify proteins according to COG and KEGG classification, respectively. COG color codes are used according to https://www.ncbi.nlm.nih.gov/COG/

Additionally we found enzymes for BChl synthesis, terpenoid backbone biosynthesis, and carotenoid synthesis. Noteworthy, elements of the reduction pathways driven by photosynthesis are shared with oxidative phosphorylation in PSB. We found in total 23 protein subunits involved in substrate respiration including the NADH dehydrogenase subunits NuoCDEFG and HoxFU, the cytochrome reductase CytB and Cyt1, seven F-type ATPase subunits and two *cbb*3 cytochrome *c* oxidase subunits. PSB use the ATP and NAD(P)H derived from photosynthesis to fix CO_2_ through the CBB cycle. For str. Cad16^T^ a complete CBB cycle with the key enzymes CbbM/RbcL RuBisCO form II (AUB81831.1) and phosphoribulokinase PrkB (AUB79979.1) were present. The fixed carbon enters the central carbon metabolism as 3-phospho-D-glycerate. In both growth conditions, str. Cad16^T^ contains enzymes for glycolysis and gluconeogenesis, as well as pyruvate oxidation, the glyoxylate cycle and the citrate cycle (TCA) in unvaried abundance. Additionally, the presence of malic enzyme (MaeB; AUB82893.1) may allow the entry of malate into the central carbon pathway via pyruvate, as shown for *A. vinosum*. In both conditions examined, the PHB synthase subunits PhaC and PhaE are expressed (AUB80707.1 and AUB84676.1). Also enzymes necessary for amino acid biosynthesis and Co-factor and vitamin synthesis were expressed under both groups analyzed.

### Proteins Differentially Expressed

In a second step, we analyzed the proteome data for significant changes between growth conditions. However, we found a large within-group variation and no proteins were significantly differently expressed between the two conditions using (paired) corrected *t*-test on transformed protein intensities (q.mod <0.01). The expression dataset was alternatively analyzed using correlation-adjusted *t*-scores (CAT scores) in order to additionally address the correlative structure of the dataset as only 4.5% of the proteins are differentially expressed. Sixty proteins were found differentially expressed (1% of all protein coding sequences) at a local false discovery rate of lfdr <0.05 (Tables 2, 3).

**Table 2:** List of “*Thiodictyon syntrophicum*” strain Cad16^T^ proteins identified as more abundant in the light period.

**Accession**	**Name**	**COG category**	**Cat score**	**Lfdr**
AUB81546.1	Cytochrome c1	C	5.44	9.53E-11
AUB83791.1	NADH-quinone oxidoreductase subunit I	C	4.35	8.32E-05
AUB84560.1	F0F1 ATP synthase subunit epsilon	C	3.51	2.26E-04
AUB83997.1	Acetolactate synthase small subunit	E	2.88	1.23E-02
AUB81260.1	Glucose-6-phosphate isomerase	G	3.63	2.26E-04
AUB83261.1	Starch synthase	G	4.48	2.53E-06
AUB79823.1	Thiazole synthase	H	2.74	2.93E-02
AUB81699.1	Dihydroneopterin aldolase	H	3.41	1.29E-03
AUB83078.1	DNA topoisomerase I	L	2.98	3.81E-03
AUB84659.1	Outer membrane lipoprotein carrier protein LolA	M	5.43	2.03E-10
AUB82245.1	HflK protein	O	6.34	1.24E-13
AUB82609.1	ATP-dependent protease ATP-binding subunit ClpX	O	3.38	1.29E-03
AUB84025.1	Molecular chaperone DnaJ	O	6.17	1.24E-13
AUB79510.1	Hypothetical protein	S	2.84	1.23E-02
AUB81403.1	Hypothetical protein	S	3.53	2.26E-04
AUB82197.1	Hypothetical protein	S	3.54	2.26E-04
AUB82687.1	Hypothetical protein	S	3.96	1.21E-04
AUB83592.1	Histidine kinase	S	3.51	1.29E-03
AUB84052.1	Host attachment protein	S	3.51	2.26E-04
AUB84690.1	Hypothetical protein	U	3.02	3.81E-03
AUB80231.1	Multidrug ABC transporter ATP-binding protein	V	3.83	1.21E-04

*COG, Cluster of Orthologous Genes; Cat, t-score for each group and feature the cat score of the centroid vs. the pooled mean (Zuber and Strimmer, 2009); Lfdr, local false discovery rate*.

**Table 3:** List of “*Thyodictyon syntrophicum*” strain Cad16^T^ proteins identified as more abundant in the dark period.

**Accession**	**Name**	**COG category**	**cat score**	**lfdr**
AUB80187.1	Isocitrate dehydrogenase (NADP)	C	2.94	3.81E-03
AUB81294.1	Oxidoreductase	C	3.54	2.26E-04
AUB82371.1	Adenylylsulfate reductase alpha subunit	C	3.03	3.81E-03
AUB83156.1	NADH dehydrogenase NAD(P)H nitroreductase	C	3.03	3.81E-03
AUB84003.1	Rubrerythrin	C	2.54	4.44E-02
AUB80125.1	Cell division protein FtsZ	D	2.67	2.93E-02
AUB79575.1	Extracellular solute-binding protein, family 3	E	2.62	4.44E-02
AUB81750.1	Decarboxylase	E	2.63	2.93E-02
AUB83785.1	4-hydroxy-tetrahydrodipicolinate synthase	E	2.94	4.18E-03
AUB84954.1	acetolactate synthase	E	2.76	1.95E-02
AUB84325.1	Proline iminopeptidase	E	2.91	4.18E-03
AUB82337.1	Adenylosuccinate synthase	F	2.54	4.44E-02
AUB79762.1	Ubiquinone biosynthesis regulatory protein kinase UbiB	H	2.86	1.23E-02
AUB80927.1	uBA THIF-type NAD FAD binding	H	2.64	2.93E-02
AUB83525.1	Synthase	I	5.36	6.66E-10
AUB81024.1	Lysyl-tRNA synthetase	J	3.31	1.29E-03
AUB79657.1	OmpA MotB	M	5.26	9.24E-09
AUB80121.1	Cell wall formation (By similarity)	M	3.04	3.81E-03
AUB83127.1	Choloylglycine hydrolase	M	2.98	3.81E-03
AUB83783.1	Conserved repeat domain protein	M	2.58	4.44E-02
AUB83066.1	Twitching motility protein	N, U	3.14	3.81E-03
AUB79946.1	Heat-shock protein	O	2.65	2.93E-02
AUB82608.1	Endopeptidase La	O	5.02	1.01E-07
AUB85620.1	DnaK-related protein	O	2.78	1.23E-02
AUB81090.1	Protein of unknown function (DUF971)	S	3.55	2.26E-04
AUB82842.1	Dinitrogenase iron-molybdenum cofactor biosynthesis protein	S	3.30	3.81E-03
AUB83253.1	Nitrogen fixation protein	S	2.96	3.81E-03
AUB83860.1	Tellurite resistance protein TehB	S	2.76	1.23E-02
AUB83943.1	Short-chain dehydrogenase reductase SDR	S	2.91	4.18E-03
AUB84318.1	Transposase	S	2.99	3.81E-03
AUB84331.1	Dienelactone hydrolase	S	2.91	4.18E-03
AUB85354.1	Protein of unknown function (DUF2868)	S	3.78	2.26E-04
AUB82296.1	Ppx GppA phosphatase	T	2.79	1.23E-02
AUB82910.1	YciI-like protein	T	2.63	2.93E-02
AUB79943.1	General secretion pathway protein G	U	2.96	3.81E-03
AUB80110.1	Hypothetical protein	–	3.21	3.81E-03
AUB81753.1	ATPase	–	4.76	1.20E-06
AUB82624.1	Hypothetical protein	–	2.81	1.23E-02
AUB82926.1	Hypothetical protein	–	2.99	3.81E-03

*Lfdr, local false discovery rate; cat, t-score for each group and feature the cat score of the centroid vs. the pooled mean (Zuber and Strimmer, 2009)*.

During the day period 21 proteins were found relatively more abundant for str. Cad16^T^. Thereof, all protein-coding sequences were annotated with eggNOG (Table 2). In presence of light, str. Cad16^T^ over-expressed four proteins involved in oxidative phosphorylation that were subunits of NADH:quinone oxidoreductase, cytochrome *bc*1 complex respiratory unit and AtpC subunit of F-type ATPase. Two enzyme involved in the central carbon pathway were abundant, the glycogen synthase GlgA and the glucose-6-phosphate isomerase GPI involved in glycolysis. The enzyme acetolactate synthase I/III small subunit associated with thiamine synthesis and the A 7,8-dihydroneopterin aldolase FolB involved in tetrahydrofolate biosynthesis were additionally found. Also membrane transport systems were found, including the ABC-2 type transport system and Twin-arginine translocation (Tat). Additionally, chaperone-type proteins DnaJ and proteolytic ClpX were also over-expressed. During the night period 39 proteins were shown to be more abundant and thereof 35 entries (89%) were successfully annotated to COG categories with eggNOG (Table 3). Analysis of this data suggested that the NAD(P)H isocitrate dehydrogenase Idh1 responsible for carbon oxidation from oxaloacetate to 2-oxoglutarate in the TCA cycle as well as a FabH 3-oxoacyl-[acyl-carrier-protein] synthase III involved in fatty acid biosynthesis initiation and elongation were abundant. The adenylylsulfate reductase subunit alpha was found, and AprA is responsible for sulfite oxidation to 5'-adenylyl sulfate (Weissgerber et al., 2014). Str. Cad16^T^ further expressed proteins associated to cell division (FtsZ), cell wall formation CpxP, Lysine and branched amino acid synthesis and nucleotide metabolism (Ppx-GppA; exopolyphosphatase and RutE; 3-hydroxypropanoate dehydrogenase). Elements of two secretion pathways were identified, a putative polar amino acid transport system and type II general secretion system. Additional three proteins detected to be more abundant in the dark are involved in stress response. The BolA-family transcriptional regulators, is a general stress-responsive regulator. Rubrerythrin may provide oxidative stress protection via catalytic reduction of intracellular hydrogen peroxide and a ATP-dependent serine protease mediates the degradation of proteins and transitory regulatory proteins, and ensures cell homeostasis.

## Discussion

We compared light to dark carbon fixation metabolism under micro-oxic conditions of PSB str. Cad16^T^ through a combination of longitudinal monitoring of physicochemical conditions, FCM counts of the microbial population, ^14^C-radioisotope uptake and quantitative proteomics of cultures incubated *in situ* in the Lake Cadagno chemocline.

Whereas the monimolimnion has been described as anoxic (Del Don et al., 2001), recurrent blooms of oxygenic plankton (Camacho et al., 2001; Danza et al., 2018) and optode-based measurements of dissolved oxygen have revealed micro-oxic conditions below 20 nM in the chemocline (Milucka et al., 2015). CTD measurements (this study) and FCM counts of the chemocline throughout the summer 2017 (Danza et al., 2018) revealed a cyanobacterial bloom down to the monimolimnion from July 2017 onward that resulted in a partly oxygenated chemocline with around 0.6 mg O_2_ L^−1^ (19 μM) below a depth of 12 m. The chemocline also retained some of the produced oxygen through the night (Figure 2). Consequently, the O_2_ present at the chemocline could possibly be used by str. Cad16^T^ as electron acceptor during mixotrophic growth, as shown *in vitro* (Peduzzi et al., 2012). In parallel, the maximal peak of turbidity related to PSB and GSB (10^6^ cells mL^−1^) sunk from around 12–14 m depth. Taken together, a dense microbial population inflicted increased self-shading below a depth of around 14 m. For this reason, we decided to lower the dialysis bags from 12 to 14 m at the 23 August 2017. Subsequently, the average light availability measured at the chemocline was 10× lower than previously recorded at 12 m (Figures 1C,D). These low light conditions at 14 m depth possibly reduced net photosynthesis and subsequent carbon storage capacity and growth for the chemocline population and for the str. Cad16^T^ incubations, as previously observed for PSB *Chromatium* spp. in the Lakes Cisó and Vilar (Montesinos and Esteve, 1984; Guerrero et al., 1985). In accordance, we found evidence for a reduced photosynthetic activity of the phototrophic sulfur bacteria in the chemocline, with medians of 757 and 587 nM h^−1^, during the day and at night, respectively (Figure 3). The measured chemocline median CO_2_-fixation rates at both conditions were below the medians at day (1,200 nM h^−1^) and at night (2,700 nM h^−1^) previously obtained for Lake Cadagno, respectively (Table 4) (Camacho et al., 2001; Musat et al., 2008; Halm et al., 2009; Storelli et al., 2013). Importantly, it was estimated that only half of the bulk carbon is fixed by anoxygenic photosynthesis in the Lake Cadagno chemocline (Camacho et al., 2001). Thereof, PSB *C. okenii* is possibly responsible for 70% of the total anoxygenic phototrophic CO_2_ assimilation (Musat et al., 2008) and the concentration was 10^5^ cells m^−1^L^−1^—equal to 31% of the total phototrophic microbes at 14 m depth — in this study. Taken together, *C. okenii* may have thereby accounted for an average of 2,023.1 amol C cell^−1^ h^−1^ in light conditions in our study, which is five-fold lower that previously observed (Musat et al., 2008). The average GSB assimilation rate with 381.2 amol C cell^−1^h^−1^ was ten-fold higher than previously observed for *C. clatratiforme* (1–30 amol C cell^−1^ h^−1^) (Musat et al., 2008). This data possibly suggest a higher CO_2_ fixation activity of the other GSB *C. pheobacteroides* (Danza et al., 2017). Oxygenic photosynthesis contributed 2,416.7 amol C cell^−1^ h^−1^ to the C-fixation, that is 10× lower than the range of values measured in the Baltic sea (Klawonn et al., 2016). However, the measured average DIC concentration was comparable to previous measurements in October 2013 (Storelli et al., 2013; Posth et al., 2017), indicating that carbon was not the limiting resource for photosynthesis. The high dark carbon fixation rate of 7,651.8 amol C cell^−1^ h^−1^ for *C. okenii* might be explained by the constitutive activity of RuBisCO form II as discussed in Ludin (2018) is close to 8,542 amol C cell^−1^ h^−1^ as estimated in Storelli et al. (2013). GSB *C. clathratiforme* were previously found to be taking up CO_2_ in the dark (Habicht et al., 2011) and the value estimated lies within the same log as in Storelli et al. (2013) with 83.7 and 8.3 amol C cell^−1^ h^−1^, respectively.

**Table 4:** Absolute carbon uptake rates of the microbial community in the Lake Cadagno chemocline.

	**Conditions**				
	**Light**		**Dark**		
**Photosynthesis**	**Oxygenic**	**Anoxygenic**		**Depth [m]**	**References**
Carbon Uptake Rates [nM h^−1^]	3,825	4,175	7,608	11.5	Camacho et al., 2001
	1,200		–	11.5	Musat et al., 2008
	85		25	12.5	Halm et al., 2009
	6,187		4,812	12.0	Storelli et al., 2013
	987		–	11.4	Berg et al., 2016
	757		587	14.0	This study

For str. Cad16^T^, the median uptake rate of 1,073.9 amol C cell^−1^ h^−1^ during the day was within the range measured for other PSB (100–30,000 amol C cell^−1^ h^−1^) (Musat et al., 2008; Storelli et al., 2013), however 10× lower compared to a previous *in situ* ^14^C-assimilation study with strain Cad16^T^ with a mean of ~12,000 amol C cell^−1^ h^−1^ (Storelli et al., 2013). The significantly higher inorganic C-uptake rate during the day compared to night-time rates suggests active photosynthesis of str. Cad16^T^ at low light intensities, as it was described for other PSB (van Gemerden, 1980). During three months incubation FCM cell counts of str. Cad16^T^ increased three-fold, leading to an estimated doubling time of 948.0 h (39.5 d) and was as such 8× longer than observed *in vitro* at room temperature (Peduzzi et al., 2012). However, these figures are not corrected for the loss of dead cells and absorbed cell aggregates to the dialysis bags. We therefore were unable to determine the exact carbon turnover and biomass accumulation during the time of incubation. However, we consider the FCM measurements to be at least representative for the time-points of sampling.

The photosynthesis apparatus was abundant in both growth conditions, as several LHC proteins were detected in str. Cad16^T^. In accordance, also the enzymatic pathways for BChl*a* and carotenoid synthesis were expressed. The central role of dissimilatory sulfur oxidation during photosynthesis is well established for PSB (Trüper, 1964) and proteins involved were found expressed in str. Cad16^T^. The proteins Sqr, Dsr and Sat were present and sulfur globules were also observed microscopically in both conditions. In str. Cad16^T^, the CsrA (AUB84364.1) seems to be the main carbon storage regulator where it was detected under both conditions. Glycolysis under mixotrophic conditions might thereby be regulated through mRNA transcription and stability as in *A. vinosum* (Weissgerber et al., 2014). We additionally found enzymes involved in the central carbon pathways TCA, glycolysis and glyoxylate cycle in unvaried abundance. Noteworthy, isocitrate lyase was found expressed, that is involved in the glyoxylate cycle, that prevents loss of CO_2_ and ensures production of NAD[P]H via TCA (Kornberg, 1959). Further, the malic enzyme was abundant, that generates oxaloacetate via malate through anaplerotic reactions without ATP (Tang et al., 2011). The *ccb*3 cytochrome *c* oxidase found in both conditions is used in aerobic respiration and additionally used for FeS oxidation and it was speculated that str. Cad16^T^ is also involved in both aerobic (Berg et al., 2016) and anaerobic cryptic iron cycling, as found for *Thiodictyon* str. F4 (Croal et al., 2004).

**Figure 5:**
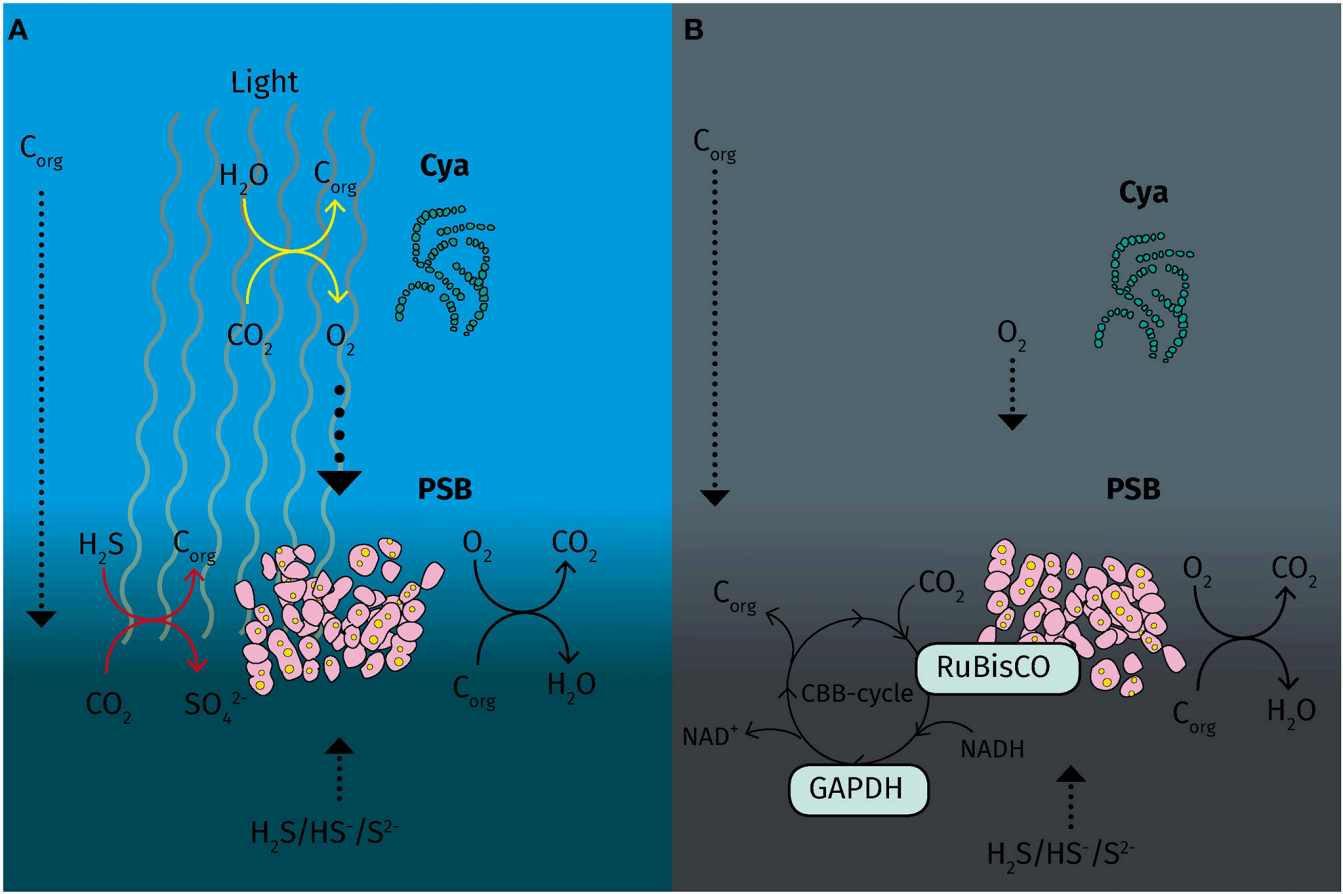
Schematic overview of carbon fixation and energy metabolism of “*Thiodictyon syntrophicum*” strain Cad16^T^ during day and night under micro oxic conditions in the Lake Cadagno chemocline. **(A)** During the day *cyanobacteria* spp. locally produce oxygen that potentially is used by PSB strain Cad16^T^ for respiration. In parallel, strain Cad16^T^ is actively oxidizing sulfide and fixing inorganic carbon in anoxygenic photosynthesis. **(B)** At night, low levels of oxygen remain in the chemocline and are used as electron acceptor in substrate respiration in strain Cad16^T^. The RuBisCO form II is possibly active in maintain the redox balance using CO_2_ as an electron acceptor. C_org_, organic carbon; Cya, cyanobacteria; GAPDH, glyceraldehyde−3-dehydrogenase; PSB, purple sulfur bacteria; RuBisCO, ribulose−1,5-bisphosphate carboxylase/oxygenase. Dotted arrows, diffusive processes; Yellow arrows, oxygenic photosynthesis; red arrows, anoxygenic photosynthesis; black arrows, chemotrophic reactions.

Under micro-oxic conditions with light, proteins involved in the oxidative respiration pathway were upregulated, indicating an active substrate respiration with light as in *T. roseopersicina* (Schaub and van Gemerden, 1994). However, the cytochrome *bc* and the NADH-dehydrogenase complex and redox carrier molecules are also used in cyclic electron transport during photosynthesis in PSB (Schaub and van Gemerden, 1994). During light conditions, both chemotrophic and phototrophic metabolism compete for electrons in str. Cad16^T^. We also found evidence for glycolysis/gluconeogenesis since glucose-6-phosphate isomerase was overexpressed. Interestingly, glycogen synthase GlgA was additionally found abundant in the light. Glycogen synthesis and sulfur oxidation was found to be ineffectively regulated in *A. vinosum* (Beeftink and van Gemerden, 1978) and we might speculate that in str. Cad16^T^ both processes are active even at slow growth, allowing for both, intracellular sulfur and glycogen accumulation. Altogether, these results indicate an active phototrophic and chemotrophic metabolism competing for electrons in the light (van Gemerden, 1980; Schanz et al., 1998). The CBB cycle is central in purple bacteria, not only for autotrophic carbon fixation, but also to regenerate the pool of reduced co-factors NAD(P)H (McKinlay and Harwood, 2010). Strain Cad16^T^ contains two forms of RuBisCO, RbcAB form I and RbcSL form II. In a previous study Storelli and colleagues (Storelli et al., 2013) detected constitutive transcription of the *rbcL* gene under autotrophic condition *in vitro* under a 12/12 h dark/light regime, whereas the form I *rbcA* was induced by light. In contrast both *rbc* genes were transcribed equally under heterotrophic conditions with acetate, with and without light, respectively (Storelli et al., 2013). In our study we detected RbcL to be equally abundant in both conditions and no other RuBisCO subunits were found. Therefore, the sole presence of the dimeric form II RuBisCO may underline the importance of CO_2_ fixation mediated by the CBB cycle possibly in maintaining the redox-balance under chemo or mixotrophic growth at low light and dark conditions, as described for purple bacteria (Laguna et al., 2010) (Figure 5).

In this study, the median dark fixation rate of str. Cad16^T^ of 834.4 amol C cell^−1^ h^−1^ was significantly lower than during the day, and around 10× lower than measured by Storelli et al. (2013). We also measured relative low total dark fixation rate in the chemocline population below the median of 2,700 nM h^−1^ (Table 4). This overall discrepancy between the studies may be explained by the ca. Ten-fold lower light availability that possibly reduced preceding photosynthetic carbon assimilation and the following dark carbon storage rates. For C-fixation at night, highly variable C-assimilation rates in chemocline bulk samples were measured, ranging from 7 to 45% of the rates measured with light (Schanz et al., 1998). Schanz et al. thereby also found a positive correlation between photosynthetically driven increase in overall biomass and subsequent dark fixation rates (Schanz et al., 1998).

In the dark, the micro-oxic condition may have led to the production of reactive oxygen species in str. Cad16^T^ that would explain the upregulated proteins involved in stress induced damage-control. Interestingly, AprA was more abundant in the night period, indicating a relative higher activity of sulfite oxidation possibly coupled to chemotrophy. Sulfur globules are consumed under dark autotrophic conditions in PSB *C. okenii* and *Chromatium minus* observed *in vitro* (Del Don et al., 1994). However, the side scatter values measured did not vary between the two sample groups in this study when monitored with FCM. This may be due to glycogen and PHB storage inclusions, structures that add up additional structural complexity and de-polymerization kinetics may be different to those of sulfur globules (Danza et al., 2017). Supporting evidence is given by the unchanged presence of enzymes involved in glycogen and PSB synthesis.

Additionally, str. Cad16^T^ possibly used the O_2_ present as electron acceptor during mixotrophic growth under both conditions as discussed above. As a consequence, some of the CO_2_ assimilated might have been constantly respired with thiosulfate as electron donor as found for *T. roseopersicina* str. M1 (de Wit and van Gemerden, 1987). In accordance, str. Cad16^T^ grew in the dark with thiosulfate and 5% O_2_ *in vitro* (Peduzzi et al., 2003). Interestingly however, we found only the SoxYZ-complex and the sulfate thiohydrolase SoxB involved in thiosulfate oxidation but no TsdA thiosulfate-oxidizing enzyme homologs encoded in the str. Cad16^T^ genome (Luedin et al., 2018a). As the complete Sox-complex is essential for the complete thiosulfate oxidation to sulfate in *A. vinosum* DSM 180^T^ (Hensen et al., 2006), str. Cad16^T^ possibly uses an alternative mechanism. One alternative may be thiosulfate uptake through the ABC transporter CysTWA (AUB80378.1, AUB80379.1, and AUB80380.1) and CysP (AUB80377.1) and oxidation to sulfite via the intermediate S-sulfocysteine by cysteine synthase B CysM (AUB82938.1) and possibly monothiol glutaredoxin of the Grx4 family (AUB83488.1) as suggested by Dahl (2017). However, with the applied methods we could not determine the relative contributions of photosynthetic or chemotrophic activity to the total increase in biomass.

## Conclusion and Outlook

PSB str. Cad16^T^ is metabolically flexible *in situ* and growths phototrophically as well as chemotrophically in the light as shown in this study. In dark conditions, low levels of oxygen may enable respiration of different small organic molecules. In summary, the 60 proteins found differentially expressed between night and day period represent only about 1% of all protein coding sequences and about 5% of the identified proteins. Therefore, their impact on metabolic pathways of str. Cad16^T^ is unclear and has to be further examined. In wider perspective, the role of PSBs in large water bodies has to be studied in more detail as we demonstrated that that low levels of oxygen can be used for respiration. As for other stratified lakes it was found that after a bloom of PSB anoxia can prevail (Bush et al., 2017), Lake Cadagno may present a more dynamic system with regular oxygenation events (Wirth et al., 2013) that infer adaption of str. Cad16^T^ to mixotrophy.

## Author Contributions

SL, NS, FD, JP, and MT conceived the study. SL, NS, FD, and SR installed the mooring and performed field measurement and sampling. NS and FD prepared scintillation samples. FD and NS did flow cytometry cell enumeration. SL extracted total protein and performed scintillation measurements. SL, MW, JP, and MT analyzed physicochemical, proteomic, and scintillation data. SL, NS, FD, and MT prepared the manuscript. All authors contributed to writing and agreed on the manuscript before review.

### Conflict of Interest Statement

The authors declare that the research was conducted in the absence of any commercial or financial relationships that could be construed as a potential conflict of interest.

## Footnotes

^1^Metabolic pathways - Candidatus Thiodictyon syntrophicum Available at: https://www.kegg.jp/kegg-bin/show_pathway?tsy01100 (Accessed June 1, 2018).

^2^Available at: http://proteomecentral.proteomexchange.org/cgi/GetDataset [Accessed June 4, 2018).
