# Supplementary material for: Mixotrophic Growth Under Micro-Oxic Conditions in the Purple Sulfur Bacterium “Thiodictyon syntrophicum”
Source: Front Microbiol. 2019 Mar 5;10:384. doi: 10.3389/fmicb.2019.00384 (PMC6413534; doi:10.3389/fmicb.2019.00384)
Supplement: Supplementary file 1 [file Data_Sheet_1.pdf]

Supplementary Material

Mixotrophic Growth under Micro-oxic Conditions in the Purple Sulfur Bacterium “*Thiodictyon syntrophicum*“

Samuel M Luedin [1 2 3*], Nicola Storelli [2], Francesco Danza [1 2], Samuele Roman [2 4], Matthias Wittwer [3], Joël F Pothier [5] and Mauro Tonolla [1 2 4‡]

[1] Microbiology Unit, Department of Botany and Plant Biology, University of Geneva, Geneva, Switzerland

[2] Laboratory of Applied Microbiology, Department of Environment, Constructions and Design, University of Applied Sciences of Southern Switzerland (SUPSI), Bellinzona, Switzerland

[3] Biology Division, Spiez Laboratory, Federal Office for Civil Protection, Spiez, Switzerland

[4] Alpine Biology Center Foundation, via Mirasole 22a, Bellinzona, Switzerland

[5] Environmental Genomics and System Biology Research Group, Institute of Natural Resource Sciences, Zurich University of Applied Sciences (ZHAW), Wädenswil, Switzerland

Correspondence:
*****Samuel Luedin
samuel.luedin@supsi.ch
‡Prof. Mauro Tonolla
mauro.tonolla@supsi.ch

Supplementary Figures

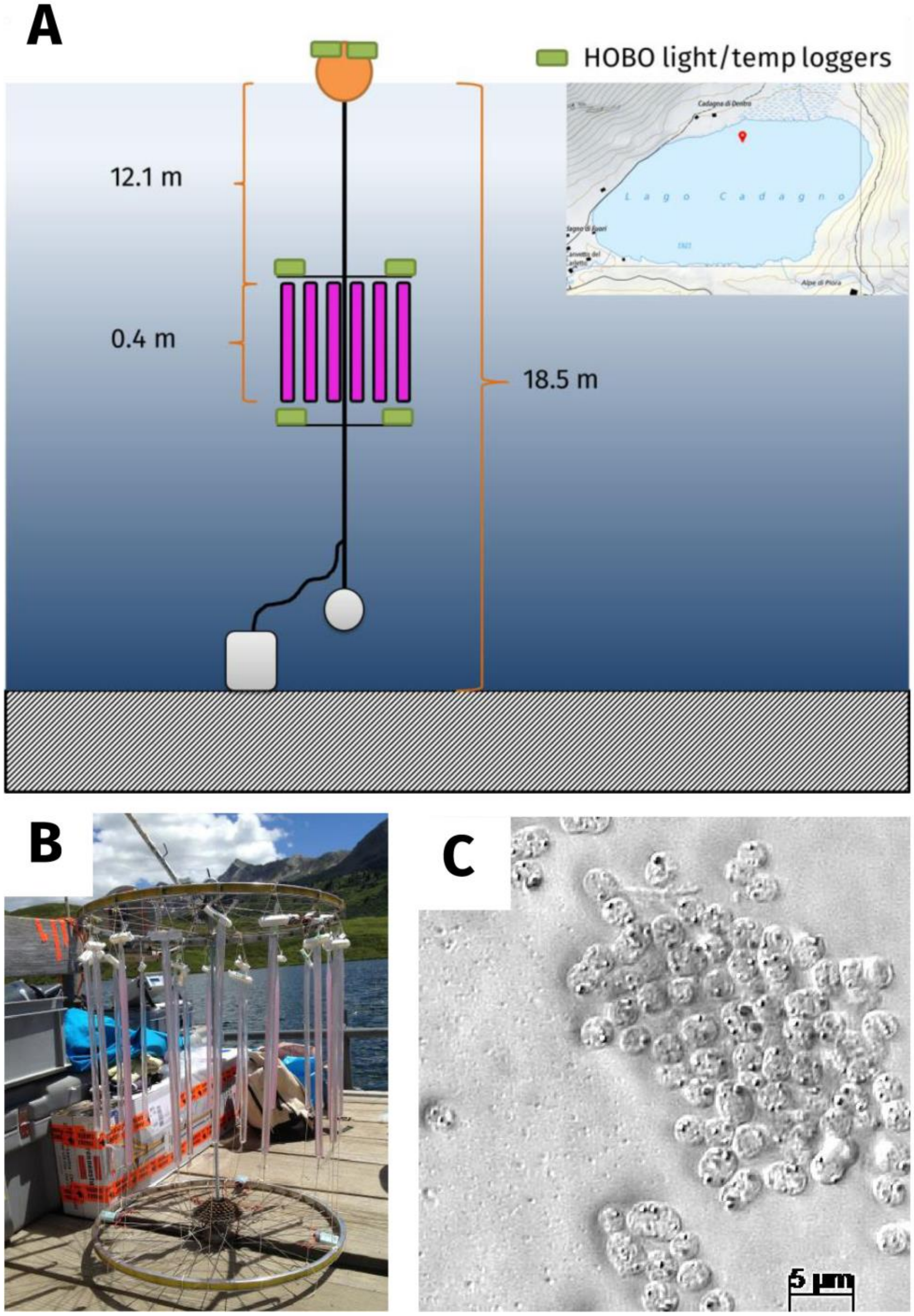

Figure S1 | Experimental setup at the sampling site and depiction of "*Thiodictyon syntrophicum*" strain Cad16^T cells A) Mooring scheme and location of the incubation experiment (initial depth, July to August) on Lake Cadagno. In pink indicated the dialysis tubes, in green the HOBO temperature and light sensors. **B)** Set-up of the strain Cad16^T cultures in dialysis tubes attached to a support grid. **C)** Phase-contrast microscopic image of pure cultures of str. Cad16^T with sulfur inclusions visible as highly refractive particles.

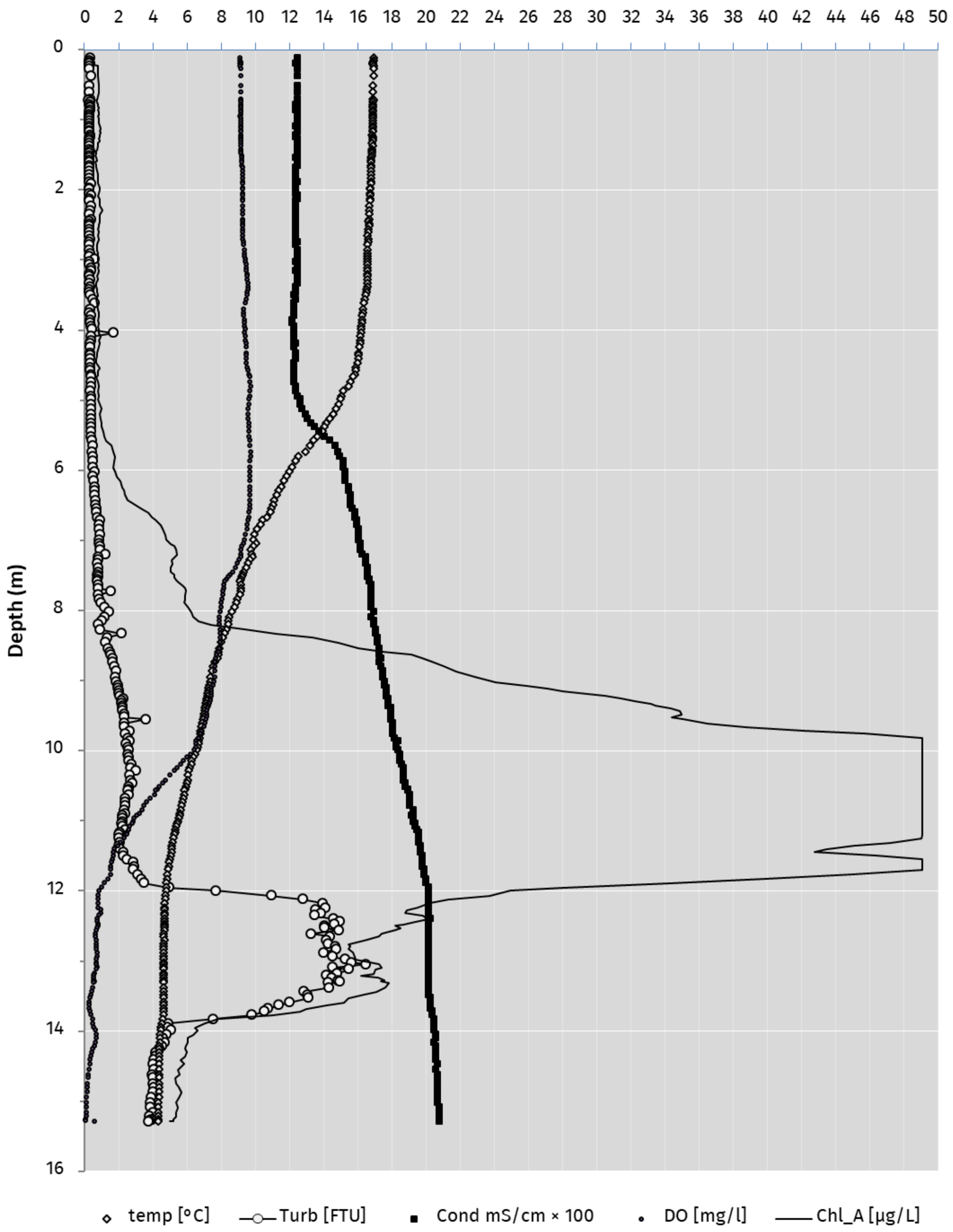

Figure S2 | Conductivity, temperature, depth (CTD) profile on July 13 2017 at 2:44 pm above the deepest point of Lake Cadagno. Chlorophyll a (—), Temperature (◊), FTU: Formazin Turbidity Unit (○), Cond: Conductivity (■), DO: Dissolved Oxygen (●),

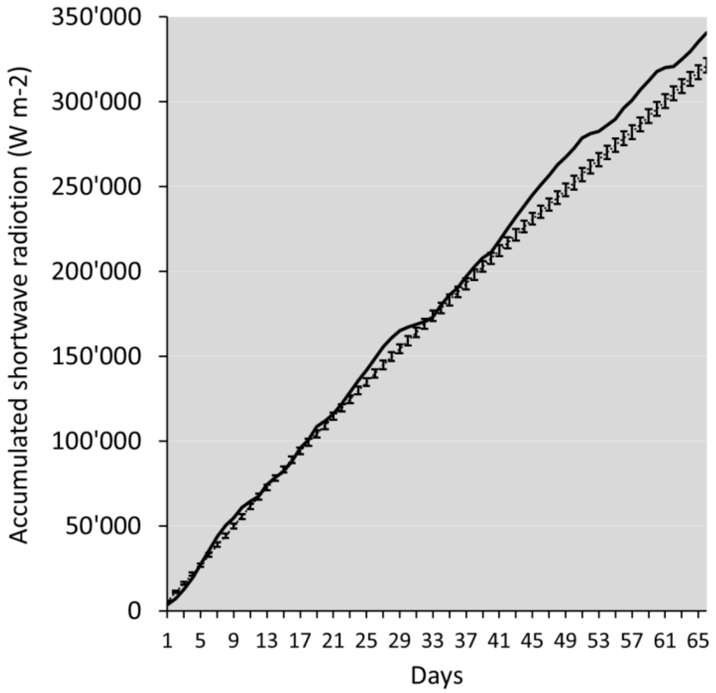

Figure S3 | Accumulated surface shortwave radiation for 66 days from 10 July to 12 September 2017 at Piora valley. The values for 2017 show above-average values (solid line). The mean values from 1985 to 2017 for the same time period are represented by the dotted line. Error bars indicate standard-error. Global radiation (diffuse and direct) on a horizontal plane given in Watt per square meter. Values derived from simulation data from meteoblue.com.

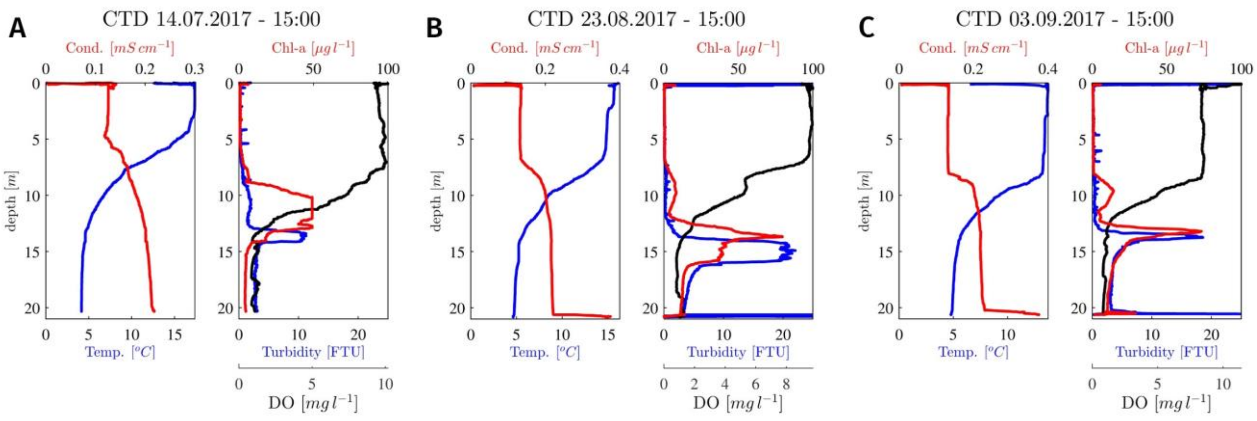

Figure S4 | Vertical conductivity, temperature, depth (CTD) profiles from Lake Cadagno from July to September 2017 at the deepest point of the lake. Temperature , conductivity, dissolved oxygen , chlorophyll a and turbidity are displayed for: **(A)** 14July 2017 **(B)** 23August 2017 and **(C)** 03 September 2017. Formazin Turbidity Unit (FTU). The data is courtesy of. Oscar Sepúlveda Steiner, Dr. Damien Bouffard and Prof. Johny Wüest. Plots are courtesy of O. Sepúlveda Steiner, APHYS Laboratory, EPFL, Switzerland.

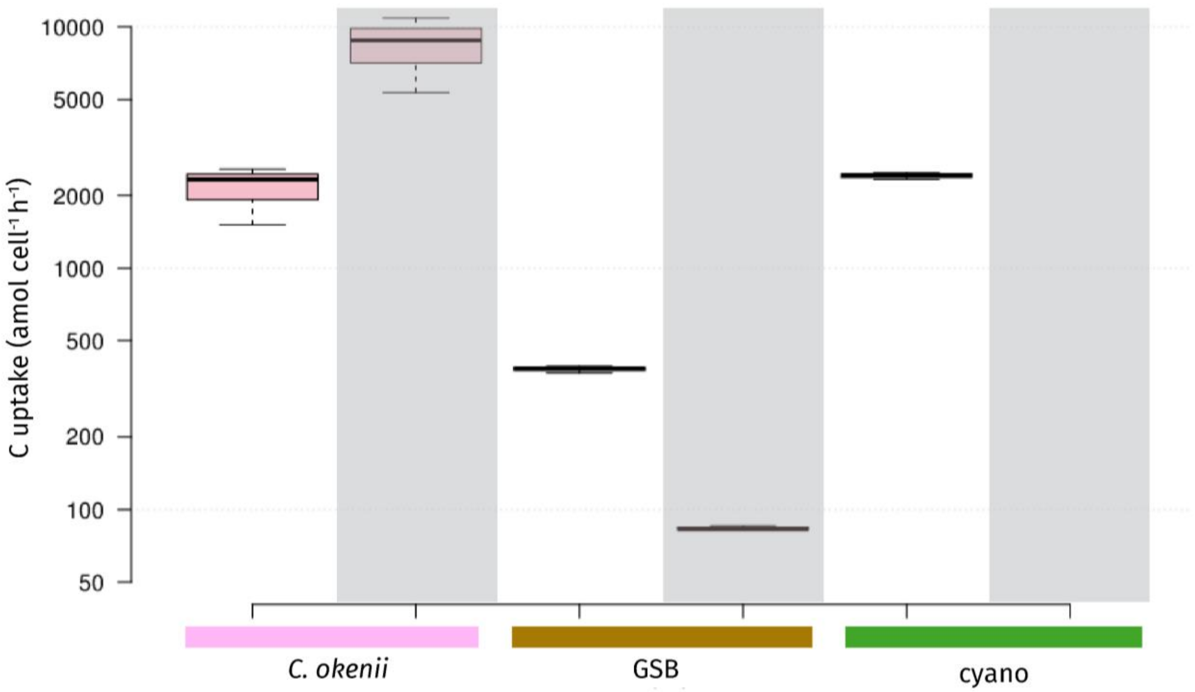

Figure S5 | Estimated average inorganic carbon uptake rates at the chemocline of the three populations counted with FCM at 1:30 pm and 9:00 pm. White background indicates profiles at 1:30 pm, grey shading indicates measurements at 9:00 pm. *Chromatium okenii* (pink), green sulfur bacteria (GSB; brown) and cyanobacteria (green). Y-axes in log scale. Center lines show the medians; box limits indicate the 25th and 75th percentiles as determined by R software; whiskers extend 1.5 times the interquartile range from the 25th and 75th percentiles, outliers are represented by dots. n = 3 sample points.
